# Parental concern and multiple dimensions of preschool children’s digital media use: a cross-sectional study

**DOI:** 10.3389/fpubh.2026.1906548

**Published:** 2026-08-10

**Authors:** Marina Ródenas-Munar, Milena Moraes, Gabriela Costa, Cristina Bouzas, Cristina Padez, Aristides M. Machado-Rodrigues, Josep A. Tur, Daniela Rodrigues

**Affiliations:** 1 Research Group on Community Nutrition and Oxidative Stress, University of the Balearic Islands-IUNICS, Palma de Mallorca, Spain; 2 CIBEROBN (Physiopathology of Obesity and Nutrition) - Instituto de Salud Carlos III, Madrid, Spain; 3 Health Research Institute of Balearic Islands (IdISBa), Palma de Mallorca, Spain; 4 Research Centre for Anthropology and Health, University of Coimbra, Coimbra, Portugal; 5 Department of Life Sciences, University of Coimbra, Coimbra, Portugal; 6 Faculty of Sport Sciences and Physical Education, University of Coimbra, Coimbra, Portugal

**Keywords:** age at first exposure, digital content, parental concern, preschool children, screen media use, socio-demographic

## Abstract

**Introduction:**

Digital media exposure is widespread during early childhood, yet the relationship between parental concern regarding children’s screen use and children’s actual digital media behaviours remains poorly understood. We examined the relationship between parental concern and multiple dimensions of children’s screen media use, including screen time, age at first exposure, and type of content.

**Methods:**

Cross-sectional data from 892 preschool children (2–6 years) recruited from the District of Coimbra, Portugal, and participating in the ScreenHealth project (2024) were analysed. Parents reported children’s screen use, socio-demographic characteristics, and concern regarding screen exposure. Screen time was categorised. Regression models were used to assess associations.

**Results:**

Concerns regarding inappropriate content, problematic or addictive screen use, and visual health were the most frequently reported by parents, whereas concerns about sleep and unhealthy food and drink advertising were less common. Moderate parental concern was associated with higher odds of children belonging to higher screen time categories (OR = 1.78, 95% CI: 1.29–2.47, *p* < 0.001) and with predominantly entertainment-oriented screen use (OR = 2.18, 95% CI: 1.28–3.71, *p* = 0.004), whereas no significant associations were observed for age at first exposure to digital media. Older child age and lower paternal educational attainment were independently associated with higher screen time categories.

**Conclusion:**

Parental concern regarding children’s screen use was associated with multiple dimensions of preschool children’s digital media use but was not consistently associated with lower screen exposure. These findings suggest that parental concern alone may be insufficient to explain or predict children’s screen use behaviours. Longitudinal studies are needed to clarify the direction of these associations. Interventions supporting healthy digital media use during early childhood should combine evidence-based guidance with practical, context-sensitive strategies that help families establish sustainable screen-use practises.

## Introduction

Digital media exposure has become an integral part of children’s everyday lives, with screen use increasingly beginning during infancy and early childhood (1, 2). Recent evidence indicates that only a minority of children younger than 5 years meet recommended screen time guidelines, with exposure levels consistently exceeding public health recommendation across many countries (2). Similar patterns have been reported in Portugal, where most preschool-aged children exceed the recommended maximum of 1 h of recreational screen time per day (3). Although digital technologies may provide opportunities for learning, communication, and entertainment, excessive or inappropriate screen use during early childhood has been associated with a range of adverse health and developmental outcomes, including poorer sleep, lower physical activity, overweight, and difficulties in cognitive, behavioural, and socio-emotional developmental (4, 5). Importantly, screen use behaviours established during the preschool years tend to persist throughout childhood (6), highlighting early childhood as a critical period for establishing healthy digital media habits.

Beyond the amount of daily screen time, growing attention has been directed towards other dimensions of children’s digital media use, particularly the age at first exposure and the type of screen content consumed. Early childhood represents a period of rapid brain development during which environmental experiences may influence language acquisition, executive functioning, social interaction, and emotional development (7). Recent cross-sectional and longitudinal studies have reported associations between earlier exposure to digital media and developmental delay, poorer language and cognitive outcomes, behavioural difficulties, and autism spectrum disorder (ASD)-like behaviours, although the available evidence remains predominantly observational and causal relationships cannot be established (8–12). In addition, the predominant purpose of screen use may also be relevant, as educational and entertainment-oriented content differ substantially in their characteristics, patterns of use, and potential developmental implications (13–15).

Parents play a central role in shaping children’s digital environments (5, 16). They regulate access to digital devices, establish screen-related routines and rules, select content, and influence the context in which digital media are used. Developmental and ecological frameworks propose that children’s behaviours emerge through continuous interactions between individual, family, and environmental factors (17–19). Accordingly, parental mediation practises, including rule-setting, monitoring, co-use, and behavioural modelling, have consistently been associated with children’s screen use behaviours (20). These parenting practises, however, are themselves shaped by broader parental perceptions, beliefs, and attitudes regarding digital media use.

Parental concern regarding children’s screen use represents one component of these broader parental perceptions. Rather than reflecting knowledge alone, parental concern may encompass emotional responses, perceived risks, previous experiences, social norms, family circumstances, or difficulties managing children’s screen use in everyday life. Recent systematic evidence indicates that parents generally recognise both potential benefits and risks of children’s digital media use. Whilst many parents acknowledge educational, communication, and learning opportunities, they simultaneously express concerns regarding excessive screen time, physical inactivity, sleep, visual health, behavioural problems, problematic use, social development, and exposure to inappropriate content (21). Parents may therefore recognise potential harms whilst simultaneously experiencing practical difficulties implementing consistent screen-related limits. Family routines, childcare demands, socioeconomic circumstances, and the widespread normalisation of digital media in everyday life may all influence the relationship between parental concern and children’s actual screen use (22–25). Importantly, recent evidence also highlights substantial heterogeneity in how parental perspectives have been measured, with relatively few studies using psychometrically robust instruments to assess parental concern or related constructs (21).

Health behaviour theories similarly suggest that recognising a potential problem does not necessarily translate into behaviour change when contextual barriers remain (22, 26). Consequently, parental concern may reflect recognition of an existing situation rather than successful management of children’s screen use. Existing qualitative and survey-based studies support this possibility, showing that many parents report concerns regarding children’s screen use whilst simultaneously struggling to implement effective restrictions in daily life (23–25). Children’s screen behaviours are also influenced by broader contextual factors including socioeconomic conditions, family routines, and lifestyle behaviours such as sleep and physical activity (27, 28). These contextual factors may further shape how parental concern relates to children’s actual digital media use.

Despite the central role of parents in children’s screen use, relatively few studies have specifically examined parental concern as an independent construct. Existing studies have primarily focused on parents’ general attitudes, beliefs, perceptions, or technology-related practises rather than directly examining parental concern and its relationship with children’s observed screen use (21). Such parenting practises have generally been associated with lower screen use amongst preschool-aged children (29, 30). One previous study reported that parents expressing greater concern about their children’s television viewing had children who watched more television, despite simultaneously reporting more restrictive home practises and attempts to regulate screen use (31). The findings suggest that parental concern may coexist with challenges in translating intentions into everyday behaviours. However, this evidence was restricted to television viewing amongst school-aged children and focused primarily on characteristics of the home environment. Whether parental concern is similarly associated with broader dimensions of digital media use during the preschool years remains largely unknown.

Therefore, the aim of this study was to examine the association between parental concern regarding children’s screen use and multiple dimensions of preschool children’s digital media use. Specifically, the objectives were to: (i) examine the association between parental concern and children’s screen time categories; (ii) investigate whether parental concern was associated with children’s age at first exposure to digital media; and (iii) examine the association between parental concern and children’s predominant type of screen use.

## Materials and methods

### Study design

This study used baseline cross-sectional data from the ScreenHealth Project, the first Portuguese longitudinal study designed to examine the impact of screen use trajectories on obesity and mental health outcomes during early childhood.^1^ Data were collected between April and June 2024 in the District of Coimbra, Portugal.

A total of 132 kindergartens and preschools were identified and invited to participate, of which 60 institutions agreed to participate (45%). Institutions that declined participation mainly reported lack of time, involvement in other projects, or concerns regarding participation in a longitudinal study. Eligible participants included preschool-aged children between 2 and 6 years and their parents or legal guardians.

Initially, 1050 children were enrolled in the study. Participants with missing data on parental concern or screen time were excluded from the analyses, resulting in a final analytical sample of 892 children. Missing values for covariates (e.g., parental education) were retained, resulting in slightly different analytical sample sizes across models.

The study was approved by the Ethics Committee of the Institute for Interdisciplinary Research of the University of Coimbra (CEIIIUC; approval date 26 July 2023) and conducted in accordance with institutional data protection procedures. Written informed consent was obtained from parents or legal guardians before participation.

Data collection included questionnaires that were completed by the child’s primary responding caregiver, most frequently the mother (74.3%), followed by the father (7.8%) and other caregivers (17.9%), including grandparents, siblings, and other legal caregivers.

### Parental concern regarding screen use

Parental concern regarding children’s screen use was assessed using six items adapted from the parental concern section of the Surveillance of digital-Media hAbits in early childhood Questionnaire (SMALLQ) (32). An additional item assessing concern regarding children’s exposure to advertising of unhealthy foods and beverages was included because of the project’s focus on obesity-related behaviours.

Respondents were asked: “*Please indicate how concerned you are about the impact that your child’s screen use may have on the following:*” sleep, visual health (e.g., eyesight), physical activity and play, exposure to inappropriate content, problematic/addictive screen use, reduced parent–child interaction, and exposure to unhealthy food/drinks advertising. Responses were recorded using a five-point Likert scale ranging from “not worried” (0) to “very worried” (4). A global parental concern score was calculated by summing responses across all items (range: 0–28), with higher scores indicating greater concern regarding children’s screen exposure. Internal consistency of the seven-item parental concern scale was high (Cronbach’s alpha = 0.92). Exploratory factor analysis using Principal Axis Factoring supported the unidimensionality of the scale (Kaiser-Meyer-Olkin, KMO = 0.912; Bartlett’s test of sphericity, *p* < 0.001). A single factor was extracted, explaining 63.9% of the total variance, with all items loading on the same factor (Supplementary Table S1). The global parental concern score was categorised into terciles (representing low, moderate, and high levels) to facilitate interpretation and to examine potential differences across increasing levels of parental concern.

### Screen media use

Respondents reported children’s screen use separately for weekdays (Monday to Friday), Saturdays, and Sundays across multiple devices, including television, video games consoles, computers, tablets, and smartphones. Device-specific durations were summed to estimate total daily screen exposure. The questionnaire did not assess simultaneous use of multiple devices; therefore, overlapping screen use could not be accounted for. Average daily screen time was calculated using the weighted formula: *[(weekday use x 5) + (Saturday use) + (Sunday use)]/7*, and expressed in minutes per day. Based on current paediatric recommendations and previous literature, screen time was categorised into three groups: low (≤60 min/day), moderate (>60–<120 min/day), and high (≥120 min/day).

Respondents additionally reported the amount of time children spent using digital devices for three purposes: educational/learning activities (e.g., reading, learning letters or numbers), entertainment (e.g., watching videos or films, listening to music, playing games), and communication (e.g., video calls with family members). Information was collected separately for weekdays and weekends. The category accounting for the greatest amount of reported screen use across the week was considered the child’s primary type of screen use. When similar amounts of time were reported across more than one category, children were classified as mixed/other. Communication use was also included in the mix category because of small representation. Respondents also reported the age (in months) at which children were first exposed to each digital device. A composite indicator representing age at first exposure to digital media was created by selecting the earliest reported exposure across devices.

### Socio-demographic characteristics

Parents reported children’s age and sex, as well as maternal and paternal educational attainment. Parental education was categorised as below university level or university degree.

### Statistical analysis

Statistical analyses were conducted using SPSS version 28.0.1 (IBM Corp., Armonk, NY, USA). Continuous variables are presented as means and standard deviations (SD), whilst categorical variables are expressed as frequencies and percentages. Multicollinearity amongst the independent variables was assessed using tolerance values and variance inflation factors (VIF). VIF values below 5 were considered indicative of the absence of problematic multicollinearity. Differences across screen time categories were examined using chi-square tests for categorical variables and one-way ANOVA for approximately normally distributed continuous variables. Analyses of individual parental concern domains were considered exploratory, and no formal adjustment for multiple comparisons was applied.

The association between parental concern and children’s screen time categories was examined using ordinal logistic regression models, with screen time category as the dependent variable. Models were adjusted for child age, sex, and parental education level. The proportional odds assumption was assessed using the test of parallel lines. A non-significant result (*p* > 0.05) was considered evidence that proportional odds assumption was satisfied. As a sensitivity analysis, the primary model was repeated using generalised estimating equations (GEE) with an exchangeable working correlation matrix and robust standard errors to account for clustering of children within educational institutions. Secondary analyses included linear regression models examining the association between parental concern scores and age at first exposure to digital media, as well as multinomial logistic regression models assessing associations between parental concern terciles and type of screen content. Educational screen use was used in the reference category. As a sensitivity analysis, the primary association between educational and entertainment-oriented screen content was re-examined using GEE with a binomial distribution, logit link, exchangeable working correlation matrix, and robust standard errors to account for clustering of children within educational institutions. Statistical significance was defined as *p* < 0.05.

## Results

The final sample included 892 preschool-aged children (52.8% boys) with a mean age of 4.7 ± 1.0 years (Table 1). The analytical sample included 32 (3.6%) children aged 2 years, 245 (27.5%) aged 3 years, 260 (29.1%) aged 4 years, 243 (27.2%) aged 5 years, and 112 (12.6%) aged 6 years. Overall, 50.3% of children were classified in the high screen time group, whilst 27.4 and 22.3% were in the moderate and low screen time groups, respectively. The mean parental concern score was 15.9 ± 8.2. Children with higher screen exposure were significantly older and more likely to have parents with lower educational attainment (*p* < 0.001 for both). Participation in organised sports was less frequent amongst children in the high screen time category (*p* = 0.035).

**Table 1:** Characterisation of the sample (*n* = 892).

		Total sample	Screen time group			*p* value
Variable	Description	n (%)	Low	Moderate	High	
Sex	Male	471 (52.8)	100 (50.3)	126 (51.6)	245 (54.6)	0.545
	Female	421 (47.2)	99 (49.7)	118 (48.4)	204 (45.4)	
Age (years)		4.7 (1.0)	4.3 (1.0)	4.7 (1.0)	4.8 (1.0)	<0.001
Screen time group	Low	199 (22.3)	–	–	–	–
	Moderate	244 (27.4)	–	–	–	
	High	449 (50.3)	–	–	–	
Global concern (score)		15.9 (8.2)	15.4 (9.7)	15.8 (8.0)	16.1 (7.6)	0.617
Concern terciles	Low	282 (31.6)	74 (37.2)	77 (31.6)	131 (29.2)	0.006
	Moderate	301 (33.7)	46 (23.1)	83 (34.0)	172 (38.3)	
	High	309 (34.6)	79 (39.7)	84 (34.4)	146 (32.5)	
Sleep (hour/day)		10.6 (1.6)	10.5 (0.6)	10.5 (1.0)	10.7 (2.1)	0.172
Organised sport	No	434 (48.8)	98 (49.5)	102 (42.0)	234 (52.2)	0.035
	Yes	455 (51.2)	100 (50.5)	141 (58.0)	214 (47.8)	
Father education	Below university	439 (52.6)	81 (43.1)	103 (44.6)	255 (61.4)	<0.001
	University	395 (47.4)	107 (56.9)	128 (55.4)	160 (38.6)	
Mother education	Below university	324 (37.2)	56 (28.7)	79 (32.8)	189 (43.4)	<0.001
	University	547 (62.8)	139 (71.3)	162 (67.2)	246 (56.6)	

Sample sizes may vary owing to missing covariate information.

**Figure 1:**
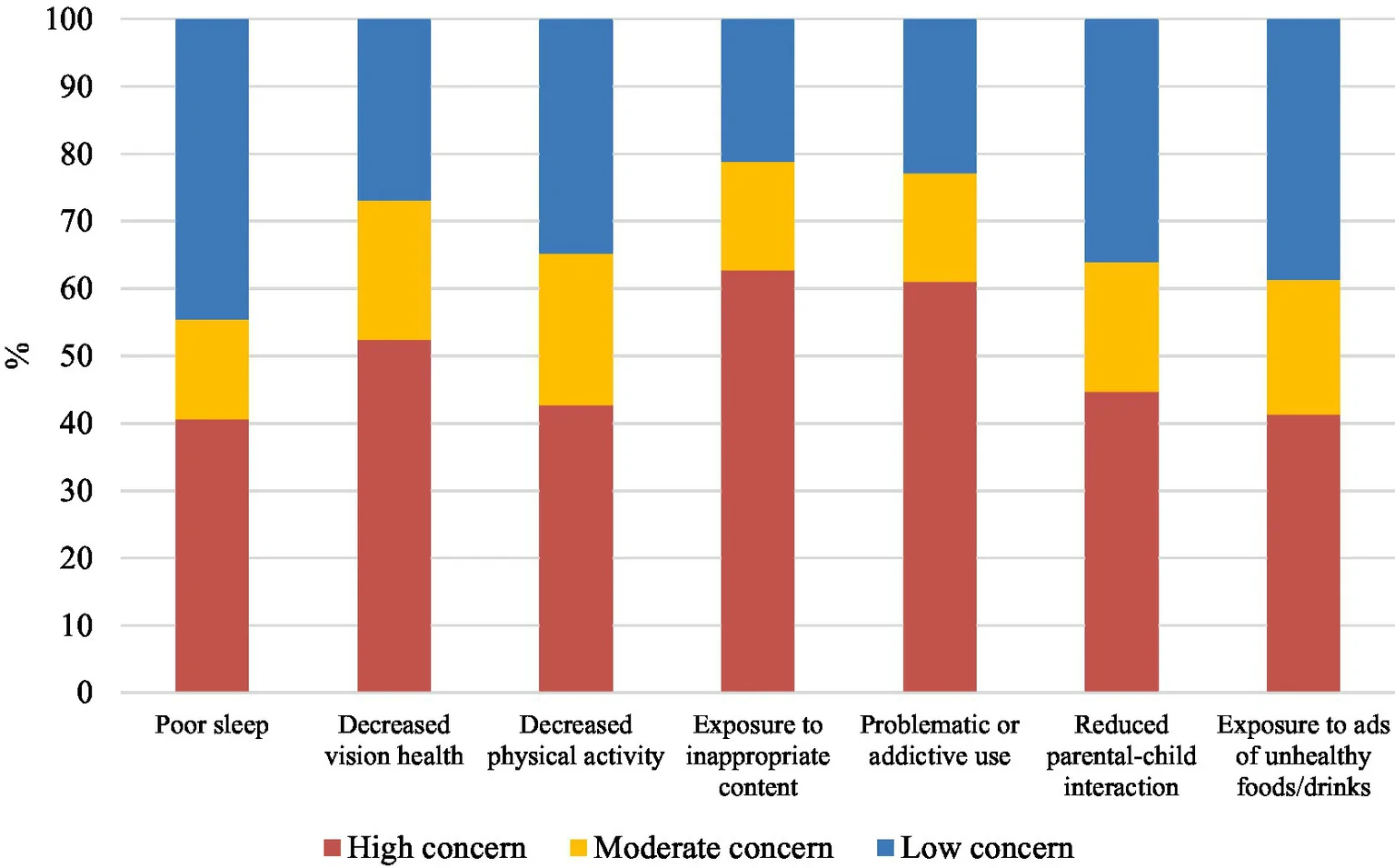
Distribution of parental concern across 7-items. Responses categories were collapsed into three levels to facilitate interpretation and ensure sufficient cell sizes, namely: High concern: worried + very worried; Moderate concern; Low concern: no + little worry.

The highest levels of concern were reported for inappropriate content (mean = 2.69 ± 1.38), problematic/addictive screen use (2.65 ± 1.38), and visual health (2.36 ± 1.51), whereas sleep (1.88 ± 1.51) and unhealthy food/drink advertising (2.02 ± 1.41) received the lowest concern ratings (Figure 1). Across screen time categories, most concern domains showed relatively similar distributions except for visual health (χ (2)=10.89, *p* = 0.028 – data not shown), where parents of children in the high screen time group more frequently reported elevated concern in this domain. However, this finding should be interpreted cautiously because no adjustment for multiple comparisons was applied and these analyses were exploratory.

The distribution of screen time categories differed significantly across parental concern levels (χ (2)=14.29, *p* = 0.006) (Table 2). A non-monotonic pattern was observed, whereby children whose parents reported moderate levels of concern showed the highest proportion of excessive screen exposure (57.1%), compared with children in the low (46.5%) and high concern groups (47.2%).

**Table 2:** Distribution of screen time categories by parental concern terciles.

Concern level	Low screen time (%)	Moderate screen time (%)	High screen time (%)	Total
Low concern	26.2	27.3	46.5	100
Moderate concern	15.3	27.6	57.1	100
High concern	25.6	27.2	47.2	100

*χ*^2^ = 14.29, *p* = 0.006.

Table 3 presents the ordinal logistic regression model adjusted for child age, sex, and parental education. Compared with the high-concern group, moderate parental concern was associated with higher odds of belonging to a higher screen time category (OR = 1.78, 95% CI: 1.29–2.47, *p* < 0.001). No significant difference was observed between the low- and high-concern groups (OR = 0.99, 95% CI: 0.72–1.37, *p* = 0.970). Amongst the covariates, older child age was associated with greater odds of belonging to higher screen time categories (OR = 1.62, 95% CI: 1.42–1.85, *p* < 0.001). In addition, children whose fathers did not have a university degree had higher odds of belonging to higher screen time categories than those whose fathers had a university degree (OR = 1.80, 95% CI: 1.34–2.41, *p* < 0.001). Maternal education and child sex were not significantly associated with screen time categories. The sensitivity analysis using GEE accounting for clustering within educational institutions produced virtually identical estimates and did not alter the statistical significance or direction of the observed associations (Supplementary Table S2).

**Table 3:** Ordinal logistic regression model examining the association between parental concern and children’s screen-time categories, adjusted for child age, sex, and parental educational attainment.

Predictor	Reference category	OR	95% CI	*p* value
Low concern	High concern	0.99	0.72–1.37	0.970
Moderate concern	High concern	1.78	1.29–2.47	<0.001
Sex	Female	1.16	0.89–1.52	0.265
Age (years)	Per additional year	1.62	1.42–1.85	<0.001
Father education	University degree	1.80	1.34–2.41	<0.001
Mother education	University degree	1.34	0.98–1.81	0.063

Model adjusted for child age, sex, maternal education, paternal education, and parental concern. Analytical sample: *n* = 824. The proportional odds assumption was satisfied (Test of Parallel Lines: *χ*^2^ = 11.06, df = 6, *p* = 0.087). Pearson goodness-of-fit indicated adequate model fit (*χ*^2^ = 1604.64, df = 1598, *p* = 0.449). No evidence of multicollinearity was observed amongst the independent variables (all VIF values ≤1.21). OR, odds ratio; CI, confidence interval.

No significant association was observed between parental concern and age at first exposure to digital media, either in unadjusted or adjusted analyses. In contrast, parental concern was associated with the type of screen content used (Table 4). Compared with children whose parents reported high concern, those with moderately concerned parents had higher odds of predominantly entertainment-oriented screen use rather than educational use (OR = 2.18, 95% CI: 1.28–3.71, *p* = 0.004). No significant associations were observed for low parental concern or for mixed/other screen use categories. Amongst covariates, older age and higher maternal educational attainment were associated with greater likelihood of entertainment-oriented screen use. Sensitivity analyses using GEE accounting for clustering within educational institutions and restricted to children with predominantly educational or entertainment-oriented screen content yielded very similar effect estimates to the primary multinomial logistic regression model. In particular, the association between moderate parental concern and entertainment-oriented screen content remained statistically significant (Supplementary Table S3).

**Table 4:** Multinomial logistic regression analysis of the association between parental concern and type of screen content.

Outcome (vs. Educational use)	Variable	OR	95% CI	*p* value
Entertainment use	Low concern	1.48	0.89–2.45	0.127
	Moderate concern	2.18	1.28–3.71	0.004
	High concern	Ref.		
	Sex	1.28	0.83–1.96	0.268
	Age	1.30	1.05–1.60	0.014
	Father education	1.49	0.93–2.40	0.098
	Mother education	1.82	1.14–2.92	0.012
Mixed/other	Low concern	0.97	0.55–1.72	0.970
	Moderate concern	1.73	0.97–3.10	0.065
	High concern	Ref.		
	Sex	1.35	0.83–2.18	0.225
	Age	1.17	0.92–1.47	0.193
	Father education	1.16	0.68–1.97	0.594
	Mother education	1.38	0.82–2.33	0.225

The high concern group was used as the reference category. Models were adjusted for child age, sex, and parental education. OR, odds ratio; CI, confidence interval.

## Discussion

This study examined the association between parental concern regarding children’s screen use and multiple dimensions of preschool children’s digital media use. Overall, parental concern was associated with children’s screen use. Specifically, moderate parental concern was associated with higher odds of belonging to higher screen time categories and with predominantly entertainment-oriented screen use, whereas no significant associations were observed for age at first exposure to digital media. These findings suggest that parental concern alone does not necessarily correspond to lower levels of children’s screen use and should not be interpreted as a direct indicator of effective parental regulation.

The distribution of individual concern domains provides additional context for understanding parents’ perceptions of children’s digital media use. Concerns regarding exposure to inappropriate content, problematic or addictive use, and visual health were the most frequently reported, whereas sleep and unhealthy food and drink advertising generated comparatively lower levels of concern. These findings are consistent with the growing body of literature showing that parents tend to hold multidimensional and sometimes competing perceptions of children’s technology use, simultaneously recognising educational opportunities whilst expressing concerns about physical health, behavioural consequences, inappropriate content, and excessive use (21). Similarly, Dardanou et al. reported that Portuguese parents commonly expressed concerns regarding technology dependence, social interaction, and negative effects on vision, whilst also recognising that digital technology had become an inevitable component of children’s everyday lives (33). Although the concern scale showed a broadly unidimensional psychometric structure, the domain-specific analyses indicate that parents assign different levels of importance to individual concerns, suggesting variation in the relative salience of specific concern domains.

Amongst the seven concern domains, only concern regarding visual health differed significantly across children’s screen time categories, with parents of children reporting higher screen exposure more frequently expressing elevated concern about vision. However, because these analyses were exploratory and no adjustment for multiple comparisons was performed, this finding should be interpreted cautiously. Nevertheless, it is noteworthy that visual health has consistently emerged as one of parents’ most salient concerns in previous qualitative and survey-based studies, including in children aged 3 to 5 years (21, 33, 34). Visual symptoms such as eye strain or concerns regarding eyesight may be more tangible and immediately observable than less visible outcomes such as sleep disturbances, behavioural changes, or socio-emotional development (35). Moreover, increasing public attention to myopia and children’s visual health following the COVID-19 pandemic may have further heightened parental awareness of this specific issue (36, 37).

The association between parental concern and children’s screen time warrants careful interpretation. Although parental concern was associated with screen time categories, the observed pattern was non-monotonic. Children whose parents reported moderate concern had significantly greater odds of belonging to higher screen time categories than those whose parents reported high concern, whereas no differences were observed between the low- and high-concern groups after adjustment for child age, sex, and parental education. Rather than indicating a straightforward protective role of parental concern, these findings suggest that concern may reflect parents’ recognition of existing screen use patterns without necessarily translating into lower exposure. Importantly, the cross-sectional design precludes any inference regarding directionality. As highlighted by the reviewers, it is equally plausible that parents become increasingly concerned after observing high levels of screen use, rather than concern preceding or determining children’s behaviour. Consequently, reverse causality represents a likely explanation and should be considered when interpreting these findings.

The present findings are also consistent with previous work by Pearson et al., who observed that parents expressing greater concern about their children’s television viewing had children who actually watched more television, despite reporting more restrictive home environments and screen-related practises (31). Together, these studies suggest that parental concern may coexist with practical difficulties in regulating children’s screen use. This interpretation is also compatible with broader health behaviour frameworks, which recognise that identifying a potential problem does not necessarily lead to successful behaviour change when contextual, environmental, or family-level barriers remain (22, 26). Another possible explanation is that coexistence of concern and screen use may reflect the complexity of balancing perceived risks with the practical role that digital media often plays in contemporary family life. Parents may recognise potential risks associated with children’s screen use whilst simultaneously relying on digital media to address practical parenting demands (e.g., education, emotional regulation) (38–41). Accordingly, parental concern should not be interpreted as a proxy for knowledge, self-efficacy, parental mediation practises, or behavioural regulation, none of which were directly measured in the present study.

Broader contextual characteristics also appeared to contribute to children’s screen use. Older children and children whose fathers had lower educational attainment showed greater odds of belonging to higher screen time categories, findings that are consistent with previous studies demonstrating socioeconomic gradients in children’s digital media use (27, 42, 43). These observations further support ecological models of child behaviour, whereby children’s screen use reflects the interaction of individual, family, and environmental influences rather than parental perceptions alone (17–19).

No significant association was observed between parental concern and children’s age at first exposure to digital media. This finding suggests that the timing of children’s first exposure to digital screens may depend less on parents’ current concern than on broader family circumstances, cultural norms, childcare practises, and the widespread availability of digital devices during infancy and early childhood. Because parental concern was measured at the time of survey completion, often several years after children’s first exposure to digital media, it may not accurately represent parents’ perceptions or family circumstances when that exposure initially occurred. Furthermore, early screen exposure is likely influenced by multiple external factors that extend beyond parents’ current concerns.

In contrast, parental concern was associated with the predominant type of screen use. Children whose parents reported moderate concern were more likely to predominantly use screens for entertainment rather than educational purposes compared with children whose parents reported high concern. Although the cross-sectional nature of the data precludes causal interpretation, this finding suggests that parental concern may relate differently to qualitative aspects of children’s digital media use than to overall screen duration. Entertainment-oriented screen use may be more visible to parents or perceived as less beneficial than educational use, potentially contributing to greater concern. However, because parental perceptions regarding content quality were not directly assessed, this interpretation remains speculative and warrants further investigation.

Taken together, these findings suggest that parental concern represents only one component within the broader set of factors influencing children’s digital media use. Rather than functioning as an isolated protective factor, parental concern appears to coexist with multiple contextual influences, including children’s age, family educational background, everyday parenting demands, and broader patterns of digital media use within contemporary family life. This interpretation is consistent with recent evidence indicating that parents often simultaneously recognise both the potential benefits and potential harms of children’s technology use (21), highlighting the complexity of decision-making surrounding digital media during early childhood.

From a public health perspective, the findings also suggest that parents may represent different profiles of concern rather than differing only in overall concern level. The predominance of concerns regarding inappropriate content, problematic use, and visual health indicates that parents prioritise some risks more than others. This observation may have practical implications for future interventions. Families reporting relatively low concern despite high children’s screen exposure may benefit primarily from evidence-based communication regarding screen use recommendations and potential health implications, whereas families expressing greater concern alongside high exposure may require practical support to implement screen-related routines within everyday family life. Because these interpretations were not directly examined in the present study, they should be considered hypothesis-generating rather than definitive. Nevertheless, they illustrate how understanding the specific domains of parental concern may help inform more tailored family-centred interventions, an area deserving future investigation.

The present findings also suggest that simply increasing parents’ concern about screen-related risks may be insufficient to influence children’s screen use. Because this study did not assess parenting practises, parental knowledge, or intervention effectiveness, no conclusions can be drawn regarding the effectiveness of specific intervention approaches. Nevertheless, the findings support the development of interventions that combine evidence-based information with practical strategies to help families establish sustainable screen-related routines, promote high-quality and developmentally appropriate digital media use, encourage parent–child co-engagement with digital media, and support alternative family activities within the realities of everyday life.

## Strengths and limitations

This study has several strengths. It included a relatively large sample of preschool-aged children and examined multiple dimensions of screen media use, including duration, content, and age at first exposure. The inclusion of both global and domain-specific parental concern measures allowed for a more comprehensive assessment of parental perceptions regarding children’s digital media use.

Several limitations should nevertheless be considered. First, the cross-sectional design precludes causal inference, and reverse causality cannot be excluded. Higher parental concern may emerge as a consequence rather than a determinant of children’s screen behaviours. Second, screen use and parental concern were assessed through parent-reported measures, which are subject to reporting bias and potential underestimation of actual screen exposure (44). The use of self-reported measures for both exposure and outcome variables also raises the possibility of common-method bias. The age at first exposure variable relied on retrospective parental recall and may therefore be affected by recall bias. Moreover, parental concern was assessed contemporaneously and may not accurately reflect parents’ perceptions, attitudes, or family circumstances at the time when children were first introduced to screens. Although most questionnaires were completed by mothers, no significant differences in parental concern scores were observed according to respondent type. Nevertheless, the predominance of maternal respondents should be considered when interpreting the findings. Third, parenting practises such as rule-setting, parental mediation strategies, and parental self-efficacy were not directly assessed. Consequently, the mechanisms linking parental concern and behavioural regulation could not be fully examined. In addition, the study did not distinguish between passive viewing and interactive screen use, nor did it assess co-viewing, parent–child interaction during screen use, or shared educational media activities. These contextual characteristics may influence the developmental implications of children’s screen exposure. Furthermore, because screen time was estimated by summing the reported duration across devices, simultaneous use of multiple devices could not be identified and may have resulted in some overestimation of total screen exposure. Another limitation of this study is that cluster-adjusted multinomial logistic regression models were not available within the statistical procedures used for the primary analyses. Consequently, clustering within educational institutions could not be incorporated directly into the multinomial model. However, a sensitivity analysis using GEE with robust standard errors was performed for the primary comparison between educational and entertainment-oriented screen content whilst accounting for clustering. This analysis yielded results that were highly consistent with the primary multinomial model, supporting the robustness of the main findings. Nevertheless, because the sensitivity analysis was based on a binary rather than multinomial outcome, it is not mathematically equivalent to the primary model and should be interpreted as complementary evidence rather than a direct replacement. Finally, the sample was drawn from a specific Portuguese region, which may limit generalizability to populations with different sociocultural and socioeconomic characteristics.

## Conclusion

Parental concern was associated with multiple dimensions of preschool children’s digital media use. In particular, moderate parental concern was associated with higher screen time and greater entertainment-oriented screen use, whereas no association was observed with age at first exposure to digital media. These findings suggest that parental concern alone may not be sufficient to explain children’s screen use and should be interpreted within the broader context of family characteristics and everyday parenting circumstances. Future longitudinal studies incorporating parental mediation practises, self-efficacy, and family routines are needed to clarify the mechanisms linking parental concern and children’s digital media use. From a public health perspective, interventions may benefit from combining evidence-based information with practical, family-centred strategies that support healthy and developmentally appropriate screen use during early childhood.

## Data Availability

There are restrictions on the availability of data for this trial, due to the signed consent agreements around data sharing, which only allow access to external researchers for studies following the project purposes. Requestors wishing to access the trial data used in this study can make a request to drdc@uc.pt.

## Footnotes

1
https://screenhealth.uc.pt
