# Supplementary material for: Parental concern and multiple dimensions of preschool children’s digital media use: a cross-sectional study
Source: Front Public Health. 2026 Aug 10;14:1906548. doi: 10.3389/fpubh.2026.1906548 (PMC13500644; doi:10.3389/fpubh.2026.1906548)
Supplement: Supplementary file 1 [file Supplementary_file_1.DOCX]

Supplementary Material

| **Supplementary Table 1.** Direct oblimin rotated component loadings for seven items. | |
| --- | --- |
| **Component** | **Factor 1** |
| Poor sleep (quality / duration) | 0.786 |
| Decreased visual health (acuity) | 0.755 |
| Decreased physical activity and play | 0.831 |
| Exposure to inappropriate content | 0.760 |
| Problematic or addictive use | 0.855 |
| Reduced parent-child interaction | 0.881 |
| Exposure to unhealthy food/drinks advertising | 0.716 |
| Eigenvalues | 4.825 |
| Percentage of total variance | 63.932 |
| *Note*: Extraction Method: Principal Axis Factoring. Kaiser-Meyer-Olkin Measure of Sampling Adequacy: 0.912. Bartlett’s Test of Sphericity: p<.001. | |

Supplementary Table 2. Generalized Estimating Equations (GEE) accounting for clustering by school (sensitivity analysis of the association between parental concern and screen time).

| **Predictor** | **Reference Category** | **OR** | **95% CI** | **p-value** |
| --- | --- | --- | --- | --- |
| Low concern | High concern | 0.99 | 0.68 – 1.47 | 0.997 |
| Moderate concern | High concern | 1.81 | 1.32 – 2.47 | <0.001 |
| Sex | Female | 1.16 | 0.90 – 1.51 | 0.256 |
| Age (years) | Per additional year | 1.62 | 1.38 – 1.92 | <0.001 |
| Father education | University degree | 1.79 | 1.30 – 2.48 | <0.001 |
| Mother education | University degree | 1.32 | 0.91 – 1.92 | 0.145 |

OR = Odds Ratio; CI = Confidence Interval. Models were adjusted for child sex, age, and parental education. Analyses accounted for clustering of children within schools using an exchangeable correlation structure and robust standard errors.

Supplementary Table 3. Sensitivity analysis using Generalized Estimating Equations (GEE) accounting for clustering within educational institutions.

| **Outcome (vs. Educational use)** | **Variable** | **OR** | **95% CI** | **P value** |
| --- | --- | --- | --- | --- |
| Entertainment use | Low concern | 1.52 | 0.91 – 2.55 | 0.110 |
|  | Moderate concern | 2.23 | 1.30 – 3.82 | 0.004 |
|  | High concern | Ref. |  |  |
|  | Sex | 0.78 | 0.50 – 1.21 | 0.266 |
|  | Age | 1.32 | 1.06 – 1.65 | 0.014 |
|  | Father education | 1.49 | 0.93 – 2.38 | 0.097 |
|  | Mother education | 1.79 | 1.10 – 2.94 | 0.020 |

Generalized estimating equation (GEE) logistic regression model restricted to children with predominantly educational or predominantly entertainment-oriented screen content (mixed/other category excluded). An exchangeable working correlation matrix and robust standard errors were used to account for clustering within educational institutions. Models were adjusted for child age, sex, and parental education. The high parental concern group was used as the reference category.
